# Biology and nature: Bionic superhydrophobic surface and principle

**DOI:** 10.3389/fbioe.2022.1033514

**Published:** 2022-10-17

**Authors:** Shangjie Ge-Zhang, Taoyang Cai, Hong Yang, Yuyang Ding, Mingbo Song

**Affiliations:** Northeast Forestry University, Harbin, China

**Keywords:** nature, bionics, superhydrophobic principle, wettability, review

## Abstract

Nature is the source of human design inspiration. In order to adapt to the environment better, creatures in nature have formed various morphological structures during billions of years of evolution, among which the superhydrophobic characteristics of some animal and plant surface structures have attracted wide attention. At present, the preparation methods of bionic superhydrophobic surface based on the microstructure of animal and plant body surface include vapor deposition, etching modification, sol-gel method, template method, electrostatic spinning method and electrostatic spraying method, etc., which have been used in medical care, military industry, shipping, textile and other fields. Based on nature, this paper expounds the development history of superhydrophobic principle, summarizes the structure and wettability of superhydrophobic surfaces in nature, and introduces the characteristics differences and applications of different superhydrophobic surfaces in detail. Finally, the challenge of bionic superhydrophobic surface is discussed, and the future development direction of this field is prospected.

## 1 Introduction

During the long evolution of the earth, it is not difficult to find that many unrelated organisms, such as lotus leaves (aquatic plants) (Yu et al., 2007; Bai et al., 2018; Han et al., 2019; Yun et al., 2020; Ghasemlou et al., 2021), roses (terrestrial plants) (Bhushan, 2018; Chen et al., 2019; Dai et al., 2019; Zong et al., 2019; Kang et al., 2021), butterflies (insects) (Qian et al., 1900; Saison et al., 2008; Wang and Guo, 2013; Bixler and Bhushan, 2014; Han et al., 2017), geckos (terrestrial animals) (Li et al., 2011; Darmanin and Guittard, 2015; Stark et al., 2016; Wang et al., 2019a; Weng et al., 2022) and sharks (fish) (Chen et al., 2018; Gose et al., 2018; Jiaqiang et al., 2018; Bilgiç and Bilgiç, 2019; Zhao et al., 2021), have evolved superhydrophobic properties. Researchers determine whether the surface is super-hydrophobic according to the contact angle of water droplets on the solid surface, that is, when the contact angle of water on the solid surface is greater than 150°, the surface is called super-hydrophobic (Huang and Guo, 2018; Shahabadi and Brant, 2019; Hasan and Nosonovsky, 2020; Hu et al., 2022). In fact, due to the difference in microstructure of each organism’s body surface, apart from superhydrophobic properties, different structures also give them different additional properties, such as self-cleaning (Dalawai et al., 2020; Wang et al., 2021a), anti-icing (Lin et al., 2018; Li et al., 2021a; Wu et al., 2021a), anti-fogging (Varshney et al., 2018; Varshney and Mohapatra, 2018; Domke et al., 2019; Fromel et al., 2021), resistance reduction (Li and Guo, 2018; Li et al., 2019) and so on. In the past few decades, superhydrophobic surfaces, as an extreme surface non-wetting state, have attracted great attention in the scientific and technological circles because of their potential applications in many fields, such as self-cleaning, anti-fouling, anti-corrosion, anti-icing and drag reduction. Inspired by these creatures, modern researchers have prepared special superhydrophobic surfaces suitable for different fields by using bionics (Ahmad and Kan, 2016; Shang et al., 2019a; Shang et al., 2019b; Wang et al., 2020a; Lin et al., 2022).

The earliest basic theory to systematically describe the phenomenon of superhydrophobic surface wetting comes from Young’s work (Young, 1805). However, in the real world, few surfaces meet the assumptions of Young’s equation, so Wenzel (uniform wetting) (Wenzel, 1949) and Cassie–Baxter (non-uniform wetting) (Cassie, 1948) respectively established new models to further improve and optimize this problem. In the later period, many scientists also put forward methods to optimize the superhydrophobic model according to different situations (Nosonovsky and Bhushan, 2005; Bhushan et al., 2007; Bittoun and Marmur, 2009; Xie et al., 2018; Jiang et al., 2020). As a hot spot in the field of material research, with the development of bionic superhydrophobic surface theory, the preparation methods of superhydrophobic surface are gradually diversified. Commonly used methods include sol-gel method (Yang et al., 2018; Vidal et al., 2019; Mahadik and Mahadik, 2021), vapor deposition method (Aljumaily et al., 2018; Pour et al., 2019; Mosayebi et al., 2020; Bayram et al., 2021; Zheng et al., 2022), etching modification method (Zhang et al., 2019a; Ma et al., 2020; Wei et al., 2021), electrochemical deposition method (Zhou et al., 2018; Xue et al., 2019a; Xue et al., 2019b; Wang et al., 2020b; Li et al., 2021b) and template pressing method (Xu et al., 2011; Victor et al., 2012). Among them, the template method can completely copy the microstructure of the biological surface, while other methods can imitate the existing structures in nature or create new structures.

In our previous review (Ge-Zhang et al., 2022), various preparation methods of bionic superhydrophobic surfaces, especially etching modification methods, were compared and described in detail. Therefore, in this mini-review, we will follow the course of human development, from using the primitive things of nature to imitating and transforming all things of nature, and then to realizing self-creation. This article focuses on the exploration and discovery of nature by human beings before self-creation. Starting from the essence, it introduces in detail the development process of superhydrophobic principle and superhydrophobic of natural organisms. This review reviews the development of superhydrophobic principle (Part 2), summarizes the structure and wettability of superhydrophobic surfaces of different animals and plants in nature (Part 3), and lists the differences and applications of different superhydrophobic surfaces. Finally, the function and application of bionic superhydrophobic surface are summarized, and the next research direction of bionic superhydrophobic surface is put forward. The current difficulties and future development directions are summarized and prospected (Part 4).

## 2 Basic principle of superhydrophobic surface

To explore the bionic superhydrophobic surface, we must first have a deep understanding of the principle. This chapter will introduce the concepts and principles of various superhydrophobic surfaces and physical models closely related to superhydrophobic properties.

### 2.1 Angle

The static wetting performance of droplets on superhydrophobic surface is usually expressed by contact angle (Voronov et al., 2008), while the rolling angle can be used to evaluate the dynamic performance of droplets on superhydrophobic surface (Hao et al., 2010).

### 2.2 Superhydrophobic model

In order to describe the relationship between the static contact angle of droplets on solid surface and the surface tension of liquid, solid and gas systems, T. Young established Young’s equation of ideal smooth solid surface state, which set a theoretical precedent for studying the wettability of materials. After that, Wensel and Cassie summarized Wensel model (Wenzel, 1949) and Cassie–Baxter model (Cassie, 1948) by studying the relationship between surface roughness and wettability, and pointed out that superhydrophobicity increased with the decrease of surface free energy and the increase of surface roughness. In modern times, more models have been optimized and pointed out (Miljkovic et al., 2013; Jiang et al., 2020; Mohseni et al., 2021; Shen et al., 2021).

#### 2.2.1 Young’s equation

**FIGURE 1:**
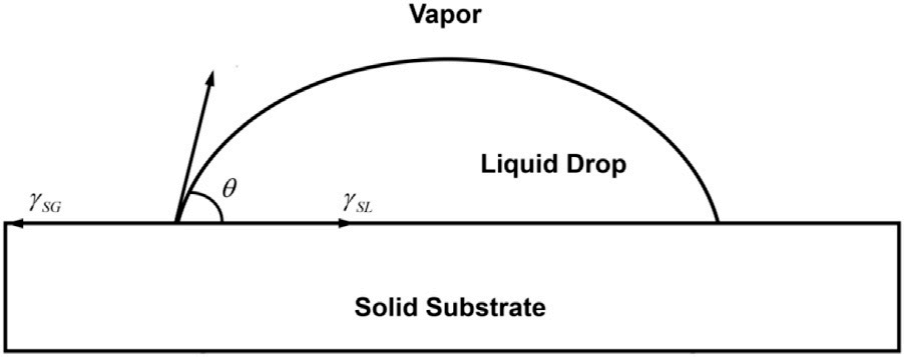
Diagram of young’s equation.

For an ideal solid surface which is uniform, smooth and rigid, Young put forward Young’s equation by means of the thermodynamic equilibrium equation in order to explain the quantitative relationship between contact angle and solid-liquid-gas interface (Figure 1):

$$\cos\theta =\frac{\gamma_{SG}-\gamma_{SL}}{\gamma_{LG}}$$

Where $\gamma_{SG}$ , $\gamma_{SL}$ , $\gamma_{LG}$ are the surface tensions between the solid-gas, solid-liquid and liquid-gas interfaces, respectively, then it is easy to know that the magnitude of the contact angle θ is jointly determined by the surface tensions of solid, liquid and gas, that is, the hydrophobic properties of solid materials increase with the decrease of their surface free energy.

However, it is found that even the smooth surface constructed by the lowest surface energy substance (fluoride) has a contact angle of only 119°, which is far lower than the superhydrophobic surface with rough surface microstructure in nature. This is because the surface roughness will also affect the contact angle. In reality, many surfaces often have a certain degree of roughness, which is not completely smooth, undistorted and uniform. Therefore, Young’s equation can only be applied to ideal surfaces, but not to realistic rough solid surfaces (Marmur, 1983). There are many modifications to Young’s equation to deal with the shortcoming that the contact angle cannot be explained and predicted for rough surfaces (White, 1977; Dobbs, 1999; Butt et al., 2007; Alizada and Sofiyev, 2011; Makkonen, 2016; Liu et al., 2020). Starov and Velarde. (2009) considered the influence of absorption liquid layer and liquid vapor, and made the following modifications and improvements to Young’s equation:

$$\cos\theta \approx 1+\frac{1}{\gamma }\int_{e}^{\infty }\Pi \left(e\right)de$$

They defined the contact angle in this case as an angle between the horizontal axis and the tangent to the droplet cap profile at the point where it touches the absorbed layer of molecules (also called the precursor film). Where e is the thickness of the absorbing liquid molecules overlaid on the solid substrate, Π(e) is the disjoining pressure. Letellier et al. (2007) considered the influence of solid liquid vapor three-phase line under the condition of system equilibrium, and established a more extensive Young’s relationship. It includes a term inversely proportional to the radius of the circle defined by the triphase line, where σ is the line tension of the three-phase contact circle:

$$\cos\theta =\frac{\gamma^{SV}-\gamma^{SL}}{\gamma^{LV}}-\frac{\sigma }{\gamma^{LV}R^{SLV}}$$

In order to further expand the application range of Young’s equation, Lin and Hong. (2019) further deduced the Young’s equation considering the contact between oil droplets and ideal smooth solid surface:

$$\cos\theta_{OW\left(Y\right)}=\frac{\gamma_{OV}\cos\theta_{OV}-\gamma_{WV}\cos\theta_{WV}}{\gamma_{OW}}$$

Among them, the underwater oil contact angle ( $\theta_{OW\left(Y\right)}$ ) is related to the interfacial tension or interfacial energy of oil-steam ( $\theta_{OW\left(Y\right)}$ ), water-steam ( $\gamma_{WV}$ ) and oil-water ( $\gamma_{OW}$ ) interfaces. The $\theta_{OV}$ is the contact angle of oil droplets in air, and $\theta_{WV}$ is the contact angle of water droplets in air.

#### 2.2.2 Wenzel model

In 1936 (Wenzel, 1949), Wenzel hypothesized that droplets in contact with a rough solid surface would produce a complete wetting phenomenon, that is, filling the grooves of the surface so that the actual contact area of solid-liquid on the rough surface is larger than the apparent contact area. Because the surface energy of rough surface is low, the contact angle of droplets is high, while the surface energy of smooth surface is high and the contact angle of droplets is low, Wenzel introduced the surface roughness (i.e., the ratio of the real surface area of the solid to the apparent geometric area, whose value is usually greater than 1):

$$\gamma =\frac{S}{S_{0}}$$

where denotes the actual surface area of the solid surface and denotes the apparent surface area of the solid surface. Then the Wenzel model can be expressed as:

$$\cos\theta_{\gamma }=\gamma \cos\theta$$

Where $\theta_{\gamma }$ is the apparent contact angle of the droplet on the rough surface, and is the intrinsic contact angle of Young’s equation. By studying the Wenzel model, the following conclusions can be confirmed: under the γ>1 usual conditions of hydrophobic surfaces, increasing the surface roughness γ will increase the apparent contact angle $\theta_{\gamma }$ of droplets under the usual hydrophobic surface conditions, which indicates that the surface hydrophobic effect will increase; For hydrophilic surface, increasing the surface roughness γ will decrease the apparent contact angle $\theta_{\gamma }$ of droplets, which indicates that the hydrophilic effect of the surface increases. This model provides a theoretical basis for the preparation of super hydrophobic surface materials. However, the applicability of Wenzel model to homogeneous solid surfaces (solid surfaces composed of homogeneous chemical substances) is still limited, and it is not suitable for heterogeneous solid surfaces, nor can it explain the phenomenon that some hydrophilic surface materials can be converted into hydrophobic surfaces after being treated (Herminghaus, 2007; Chen et al., 2021a). At the same time, under the assumption that the droplets are completely wetted, the large energy barrier formed by the chemical composition and geometry will make it difficult for the droplets to roll. This contradicts the phenomenon that droplets are easy to roll on the superhydrophobic surfaces such as lotus leaves in nature (Nuraje et al., 2013; Rius-Ayra et al., 2018).

Seo and Kim. (2015) derived the modified Wenzel equation by considering the constant volume of droplets as an auxiliary condition and a transverse condition:

$$\cos\theta =K\frac{\gamma_{SO}-\gamma_{SL}}{\gamma }=K\cos\theta_{Y}$$

where $\theta_{Y}$ is the equilibrium contact angle on a smooth solid surface and θ is an apparent.

In order to solve the problem that the Wenzel model is only suitable for ordered arrays or uniform porous media with uniform characteristics, Han et al. (2007) proposed a modified Wenzel model to describe heterogeneous surfaces as follows:

$$\cos\theta =\frac{\cos\theta }{S}\int_{X_{\min}}^{X_{\max}}\frac{W_{0}}{\delta \sqrt{2\pi }}\frac{1}{X}\left[-\frac{{\left(X_{0}-X\right)}^{2}}{2\delta^{2}}\right]dX$$

where W is the cumulative micropore volume, $W_{0}$ is the totalmicropore volume determined from the D–R equation, $X_{0}$ is themicropore half width at the distribution curve maximum, and δ is the dispersion parameter.

#### 2.2.3 Cassie–Baxter model

In 1944, Cassie and Baxter (Cassie, 1948) considered the influence of surface tension, and put forward the concept of compound contact. Because the size of the roughened surface structural unit is smaller than that of the droplet, the droplet on the surface can not completely penetrate into the groove on the surface, which results in air staying in the groove. Therefore, the Cassie–Baxter model of solid-liquid-gas three-phase composite contact is established:

$$\cos\theta_{c}=f_{1}\cos\theta_{1}+f_{2}\cos\theta_{2}$$

where $\theta_{c}$ is the apparent contact angle of the droplet on the rough surface, $\theta_{1}$ and $\theta_{2}$ are the intrinsic contact angles on the two media, $f_{1}$ and $f_{2}$ are the proportional fractions of the solid-liquid and air-liquid contact surfaces at the composite interface, respectively, and ${f_{1}+f}_{2}=1$ . Because the inherent contact angle between droplet and air is 180°, the model can be simplified as follows:

$$\cos\theta_{c}=f_{1}\cos\theta_{1}-f_{2}$$

**FIGURE 2:**
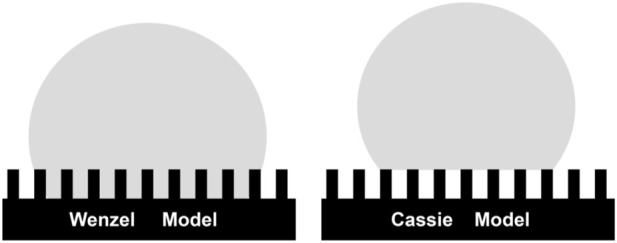
Microscopic diagram of droplets on the Wenzel model (left) and Cassie–Baxter model (right).

From this model, it can be clearly seen that the smaller the solid-liquid contact area ratio, the larger the contact angle of the rough surface and the better the hydrophobicity. This model explains some phenomena, for example, droplets on super-hydrophobic surfaces such as lotus leaves and rice leaves show very small rolling angle and hysteresis angles, which is difficult to be explained by Wenzel model. Figure 2 shows the difference between Wenzel model and Cassie–Baxter model.

It is worth noting that Wenzel model and Cassie–Baxter model have their respective applicable scopes. The Cassie–Baxter model is applicable to the highly hydrophobic region where the surface adhesion force is small, while the Wenzel model is applicable to the moderately hydrophobic region where the surface adhesion force is large. As a practical matter, if the droplet overcomes the energy barrier between the two modes and reaches the corresponding energy state under the action of an external force, its wettable viscous state can be transformed between the two models. That is, the wetting state of a droplet on a rough solid surface may then be transformed between both Wenzel and Cassie–Baxter.

**FIGURE 3:**
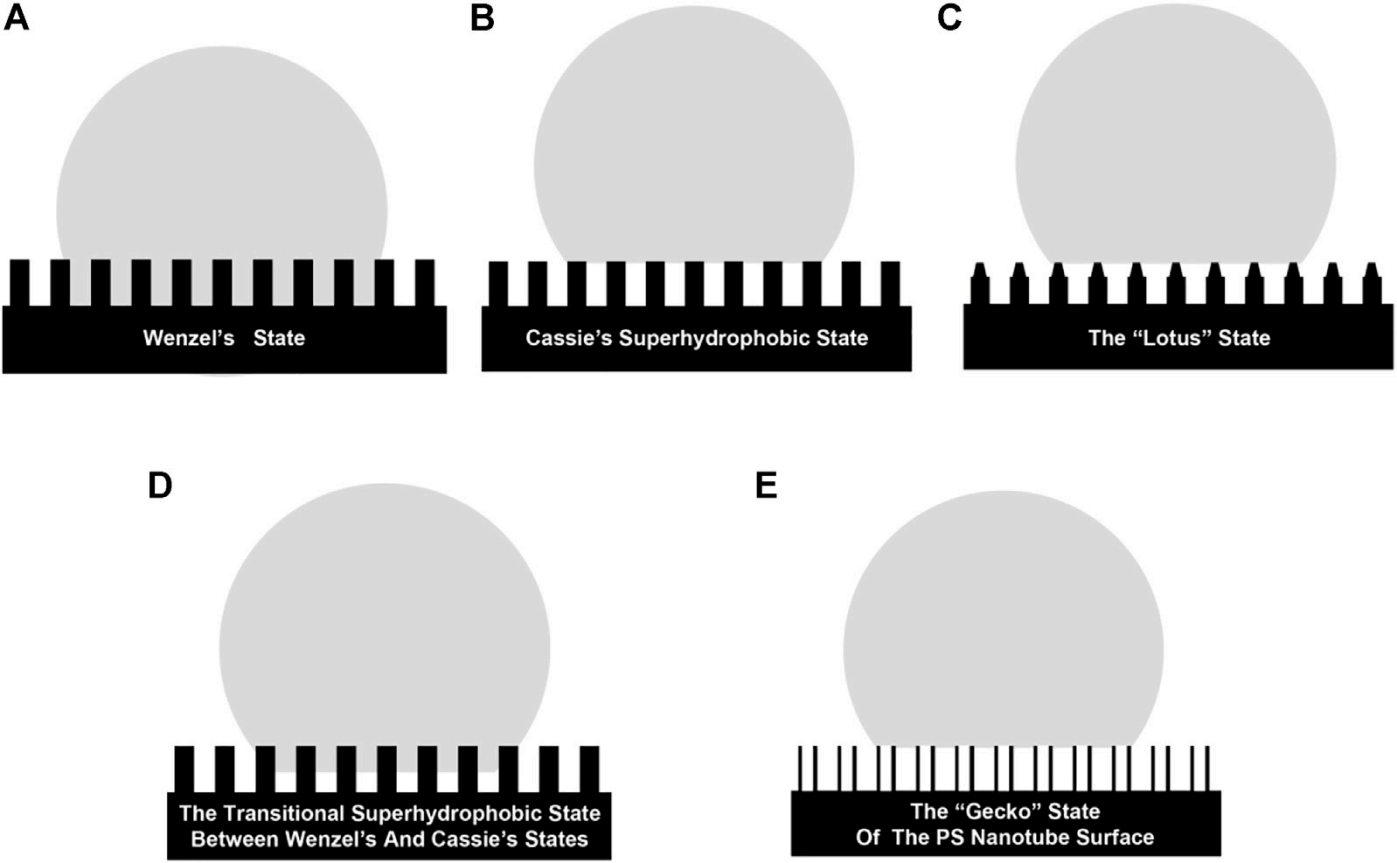
Diagram of the existence state of droplets on five superhydrophobic surfaces: Wenzel’s state **(A)**, Cassie state **(B)**, Lotus state **(C)**, Wenzel- Cassie transition state **(D)**, Gecko state **(E)**.

In addition, Wang and Jiang. (2007) further refined the existence of five superhydrophobic surfaces based on the previous work (Figure 3): the Wenzel state (droplets are embedded on the surface in a fully wetted state and contact angle hysteresis can be observed), the Cassie state (droplets are independently in contact with the surface in a non-wetted state, with low surface adhesion and easy roll-off), the Lotus state (Cassie state special case, similar to the microscopic raised structure on the surface of lotus leaf, which is important for the design and construction of bionic superhydrophobic surfaces with self-cleaning properties), the Wenzel- Cassie transition state (the state that mainly exists in reality), and Gecko state (the state where droplets on polystyrene nanotube films have extremely high surface adhesion). Ideally, the contact angle of the droplet in Wenzel state is close to 0°, while droplets in the Cassie state would form perfect spheres (ignoring gravity), with the contact angle close to 180°.

## 3 Nature's biological superhydrophobic surfaces

Through 3.7 billion years of evolution and species selection, most of the creatures in nature have survived with various unique biological functions and structures, which enable them to quickly adapt to changes in the ecosystem and surrounding environment. According to the order of research objectives in the history of superhydrophobic surface development, this chapter follows the order from plants to animals, and lists many surface structures and multifunctional applications. In addition, according to the relationship between the multifunction of superhydrophobic surfaces from simple to complex, the representative examples of plants and animals are introduced in detail.

### 3.1 The surface structures of typical plants

Table 1 lists the superhydrophobic phenomena and characteristics of many plants in nature. In fact, the first study of superhydrophobic surface by human beings started with the structure of plant surface. From dust and dirt on lotus leaves easily taken away by dew and rain, to small water drops firmly attached to rose petals on the surface, to water drops on rice leaves easily rolling towards the growth direction of leaves, natural plants have inspired us in many aspects.

**TABLE 1:** The surface structures of typical plants.

Plant surface	Properties	References
Lotus leaf	Superhydrophobic, self-cleaning, low adhesion	Cheng and Rodak. (2005); Teodorescu. (2014); Khandavalli et al. (2018); Xu et al. (2021a)
Rose petal	Superhydrophobic, high surface adhesion	Feng et al. (2008); Bhushan and Her. (2010); Lai et al. (2019)
Sunflower	Superhydrophobic, high surface adhesion	Hoefnagels et al. (2007); He et al. (2018); Liang et al. (2020)
Rice leaf	Superhydrophobic, directional transport	Wu et al. (2011); Lian et al. (2019)
Nepenthes	Directional transport, water harvesting	Wong et al. (2011); Zhang and Xu. (2021)
Purple setcreasea	Double-sided superhydrophobic	Guo and Liu (2007); Wolfs et al. (2013); Cai. (2019)
Watermelon leaf	Single-order scale hydrophobic structure	Zhang et al. (2012a); Gou and Guo. (2018); Sharma. (2021); Behera. (2022)
Peanut leaf	Superhydrophobic, high surface adhesion	Yang et al. (2014a); Yang et al. (2014b); Long et al. (2015)
Bamboo leaf	Anti-icing, high surface adhesion	Yuan et al. (2014); Zhang et al. (2019b); Gao et al. (2020)
Taro leaf	Superhydrophobic, self-cleaning	Verbanic et al. (2014); Kumar and Bhardwaj. (2020); Wu et al. (2020); Pieniazek et al. (2021)

#### 3.1.1 Lotus leaves

The lotus leaf was described by the ancient Chinese as “dirt-free plant rising from soil”, which is the most typical super-hydrophobic surface of plants (Latthe et al., 2014), and it is also one of the earliest research goals of human beings, which is why the “lotus leaf effect” is still synonymous with superhydrophobic characteristics. Later, Jiang et al. (Barthlott and Neinhuis, 1997) determined that the surface of lotus leaves is a hierarchical structure formed by micron-sized papillae and nanoscale wax crystals covering the surface, and they also explained the relationship between superhydrophobicity and self-cleaning. It is worth mentioning that in the water condensation experiment, water is hydrophilic on lotus leaves that have experienced water condensation, which shows that lotus leaves can be hydrophobic or hydrophilic, depending on how the water reaches their surface (Cheng and Rodak, 2005). Considering the characteristics of the lotus leaf and the bionic means of scientists, it has a rich and broad application prospect in production and life (Table 2).

**TABLE 2:** Bionic product with lotus leaf as template.

Main materials	Technologies	Advantages	Ref
poly-ε-caprolactone	Needle-free electrospray	Adhesion resistance	Klicova et al. (2022)
Hexamethyldisiloxane	Electrospinning		
	Plasma-assisted chemical vapor deposition		
Zinc oxide	Chemical vapor deposition	No need for metal catalysts	Li et al. (2008)
Porous anodic			
Alumina			
Perchloric acid			
Ethanol			
Acetone			
Aniline		Favorable surface-to-volume ratio	
4.4- diaminodiphenylamine sulfate hydrate	Nano casting technology	Excellent anticorrosion performance	Chang et al. (2013)
4'-(4.4′- isopropylidene-diphenoxy) bis (phthalic anhydride)		Good electrical activity, mechanical properties and high temperature stability	
N,N- dimethylacetamide			
Ammonium persulfate			
Dimethyl silicone polymer			
Rubber sponge	Ultrasonic treatment	High temperature stability	Wang et al. (2021b)
Tetrahydrofuran		Stable conductivity, high compression ratio and linear working range	
MWCNTs		Excellent sensing stability and durability	
		Self-cleaning	
Copper foil	Chemical deposition method	Adhesion resistance	Wu et al. (2014)
Silver nitrate		Strong mechanical properties	
Octadecyl mercaptan			
Fresh lotus leaves			
Polymethylhydrosiloxane			
Phenyl substituted silica	Casting method	Multi-purpose	Nagappan et al. (2013)
Ammonium hydroxide solution	Dip coating method	Environmentally friendly	
Oxalic acid	Solvent evaporation method	Low price	
Polyvinylidene fluoride		Good thermal stability and mesoporous structure	
Polytetrafluoroethylene			
Medical gauze	Physical deposition	Good blood compatibility	Li et al. (2020a)
Dopamine	Chemical deposition	Adhesion resistance	
Perfluorocarbon		Antibacterial	
Silver nanoparticles			
Alumina film	—	Good blood compatibility	Mao et al. (2009)
Sodium hydroxide		Anti-platelet	
		Anti-blood cell adhesion	

One of the more common is the application of lotus leaf in the medical field (Lim et al., 2013; Yang et al., 2014c; Wu et al., 2021b; Huang et al., 2022). Klicova et al., (2022) developed a biocompatible nanofiber pad with anti-adhesion surface by imitating the nanostructure on the lotus leaf by using needle-free electrospraying and polycaprolactone electrospinning technology, which not only shortens the operation time but also greatly reduces the postoperative risk. At the same time, inspired by the self-cleaning characteristics of lotus leaves, Li et al. (2020a) developed a new type of anti-adhesion and antibacterial gauze through three simple dipping steps. With its excellent anti-adhesion and bactericidal activity, it can promote infectious wound regeneration and meet clinical needs. Due to the increasing demand for blood compatibility of biomaterials, Mao et al. (2009) focused on the preparation of an anticoagulant biomaterial-polystyrene nanotube film, which can prevent thrombosis and tissue capsule, and is of great significance in organ transplantation. In addition, the application of super hydrophobicity of biomimetic lotus leaf is also involved in the field of gas sensors (Li et al., 2008) and meteorology (Wang et al., 2021b).

#### 3.1.2 Rose petals

In contrast to the lotus leaf, the rose petal is the canonical example in the Wenzel model. As its petal fibers have a micro-nano double-order structure scale larger than that of the lotus leaf surface, the droplets tend to completely wet the larger scale surface grooves, resulting in increased surface roughness, high surface adhesion, and strong contact angle hysteresis. This shows that even if the petals are inverted, the droplets on the surface will not fall off. Jiang et al. first discovered this phenomenon in 2008 and called it the “petal effect” (Feng et al., 2008). Subsequently, Zheng et al. (2019) studied the dynamic wetting law of viscous superhydrophobic substrates for the first time by comparing and analyzing simple artificial petal-like substrates and superhydrophobic substrates. As shown in Table 3 is bionic product with rose petals as template.

**TABLE 3:** Bionic product with rose petals as template.

Main materials	Technologies	Advantages	Ref
Polyurethane	A combination of replica molding and hydrophobic particle deposition	Reversibly transformed between Cassie–Baxter state and Cassie immersion state	Shao et al. (2020)
Polycaprolactone glycol-400; 4-butanediol			
Triethylamine			
Dimethylformamide			
4-diphenylmethane diisocyanate; Nano-fumed hydrophobic silica; Glycerol			
Dimethylol propionic acid	Template method	Real-time wetting and adhesion behavior changes in response to magnetism	Drotlef et al. (2014); Chen et al. (2021b)
PDMS			
NdFeB			
CIPs			
Red rose petals	One-step solvothermal method	Strong mechanical properties	Yang et al. (2019)
Acetone	Nanoimprint lithography method	High thermal stability	
Polyvinyl butyral		High buoyancy	
Polydimethylsiloxane			
Curing agent			
Octadecyl trichlorosilane			
Anhydrous ethanol			
Ethyl silicate			
GO			
Latex balloon; (heptadecafluoro-1.1,2,2-tetrahydrodecyl) trichlorosilane	3D shrinking method	Tunable adhesion (39.2–129.4 µN)	Tan et al. (2019)
		Ultralarge liquid capacity	
Cuprum	Chemical etching method	Simple, fast, cheap	Bahrami et al. (2017)
FeCl_3_		Controllable adhesion	
Stearic acid	Two-step molding process	Controllable adhesion	Bhushan and Her, (2010)
Rose petals	Wax evaporation method			
Chloroform; n-hexadecane			
Cuprum	Electrodeposition method	Excellent stability and corrosion resistance	Liu et al. (2014)
Hydrochloric acid		Fast and easy	
Sodium hydroxide		Low cost	
Cerium myristate			

It can be predicted that the self-cleaning functional surface with the “lotus leaf effect” has played an important role in drag reduction, cell culture, dust control (Nosonovsky and Bhushan, 2009; Ueda and Levkin, 2013), while the application prospect of “petal effect” is much broader for non-destructive fluid transfer and biotechnology (Sun et al., 2005; Lai et al., 2013; Yue et al., 2020).

It is worth noting that because the super-hydrophobic rose petals have different surface microstructure and nanostructure, the adhesion of different petals is also different. On the basis of studying two kinds of super-hydrophobic rose petals with high and low adhesion, Bhushan and Her. (2010) prepared artificial super-hydrophobic surfaces with high and low adhesion by wax evaporation, in which the droplets with high adhesion will not fall when the substrate is vertically inclined or inverted.

In addition, since rose petals and lotus leaves are natural examples of the Wenzel-Cassie transition state and the Cassie–Baxter model, respectively, an increasing number of scholars have compared the two with the intention of exploring the relationship and transition between them (Zhang et al., 2012b). The researchers realized the reversible transition between the Cassie–Baxter state and the Cassie impregnation state of the superhydrophobic surface by adjusting the micro/nanostructure of the shape memory polymer SMP. This surface controls the adhesion behavior of liquids and has an important impact on rewritable patterns and the transport and collection of controlled droplets (Shao et al., 2020). In order to apply the superhydrophobic surface to droplet microfluidic chip and microfluidic transmission, Drotlef et al. (2014) Chen et al., 2021b) focused on the magnetic response surface, and proposed a magneto rheological elastomer superhydrophobic surface with magnetic response, which can be quickly and reversibly replaced between “lotus effect” and “rose petal effect”. For large general conductor materials, Liu et al. (2014) developed a one-step electrodeposition method to prepare controllable superhydrophobic surface with excellent stability and corrosion resistance.

#### 3.1.3 Rice leaves

Compared with the former two, rice leaves show another interesting new feature: by macroscopic observation, droplets on rice leaves are easier to slide down in the growth direction of rice leaf (from the stem to the petiole or from the stem to the tip). Microscopically, the surface of rice leaves is also a super hydrophobic surface suitable for the Cassie–Baxter model, but the arrangement of its surface structure is quite different from that of lotus leaves and rose petals. Micro-nano double-stage structures are arranged orderly along the growth direction of rice leaves, but randomly in the vertical direction (Bhushan et al., 2009; Wu et al., 2011), just like the roof tile structure in ancient China. The geometric structure of micro-grooves arranged in order along the same direction makes the energy barrier overcome by liquid droplets rolling along the parallel direction of leaves and stems much smaller than the energy barrier perpendicular to the direction of leaves and stems, resulting in anisotropy of surface adhesion. The rolling angles measured by experiments are 3°–5° along the direction parallel to leaves and stems and 9°–15° in the vertical direction (Feng et al., 2002). As shown in Table 4 is bionic product with rice leaves as template.

**TABLE 4:** Bionic product with rice leaves as template.

Main materials	Technologies	Advantages	Ref
poly [6-(4-methoxy-4′-oxyazobenzene)hexyl methacrylate]	Reverse Breath Figure	Effective and convenient water collection	Gao et al. (2018)
Gold nanoparticles	Self-assembly		
1H,1H,2H,2H-perfluorodecanethiol			
Aluminum	Femtosecond laser grating scanning	Fast	Yang et al. (2021)
		Anisotropic superhydrophobic	
		Self-cleaning	
TiO_2_	3D printing technology of stereolithography	Drag reduction	Barraza et al. (2022)
Hexadeciltrimethylsiloxane		Anisotropy	
Samples of each of the rice leaf, butterfly wing, rainbow trout fish scales, and Mako shark skin	Template method	Self-cleaning	Bixler and Bhushan, (2012)
Liquid platinum silicon		Drag reduction	
Isopropanol			
Liquid carbamate polymer			
Green rice leaf			
Polydimethylsiloxane	Femtosecond laser method	Three-dimensional anisotropy	Fang et al. (2018)
Fluoroalkyl silane			
Silicon substrate			
Dimethyl siloxane			
Heptafluorodecyl trimethoxysilane	Laser etching method	Switchable isotropy-anisotropy	Cheng et al. (2018)
Bisphenol A diglycidyl ether	Chemical etching method		
N-octylamine	Template method		
M-xylylenediamine			

With the intensive study of the unique anisotropic (also called liquid-oriented) superhydrophobicity of rice leaves, once again, the field of liquid-oriented drag reduction, water collection and transport has been promoted (Gleiche et al., 2000; Higgins and Jones, 2000; Chen et al., 2005).

Therefore, the researchers are committed to constructing an anisotropic hierarchical structure based on the unidirectional sliding of water droplets in rice leaves (Zhang et al., 2012b; Gao et al., 2018; Xu et al., 2020). Yang et al. (2021) transformed the bionic superhydrophobic surface from isotropic to anisotropic by laser grating scanning, and obtained an anisotropic superhydrophobic aluminum surface with rice leaf shape. Inspired by the microstructure of lotus leaf and rice leaf, Cheng et al. (2018) proposed a new functional material. By repeatedly controlling the surface microstructure shape between lotus leaf structure and rice leaf structure, the reversible transition between isotropic and anisotropic wetting state of superhydrophobic was realized. In addition, the superhydrophobic surface has good stability, even after 1 month, intelligent transformation can be observed, and it is widely used in controlled droplet transportation. In order to highly reproduce the surface structure of rice leaves, Fang et al. Fang et al. (2018) used two-step soft transfer to develop the structure of artificial rice leaves. The structure has the sliding characteristic of anisotropy clearly. The systematic measurement shows that the sliding angles of the structure parallel to the vein direction and perpendicular to the vein direction are 25° and 40° respectively, which can be used for the rapid fabrication of large area artificial rice leaf surface without expensive instruments and complex techniques.

#### 3.1.4 Chapter summary

By comparing the superhydrophobicity of plant surface, it can be easily found that small differences in surface morphology or characteristic size will lead to great differences in surface wetting behavior. For example, the microstructure of rose petals has a larger distance than lotus leaves, which brings a completely different phenomenon, and the micro-morphology of rice leaves arranged regularly will limit the rolling direction of droplets, and so on. Therefore, when constructing and preparing superhydrophobic biomimetic materials, researchers often not only take one organism as a reference, but also combine different structures of various organisms according to the target field to achieve the purpose of meeting the application requirements.

### 3.2 The surface structures of typical animals

Plants are not the only creatures with superhydrophobic properties. Superhydrophobicity can also be found in different animals, some of which are listed in Table 5, and typical ones will be selected to be elaborated in more detail.

**TABLE 5:** The surface structures of typical animals.

Animal surface	Properties	References
Gecko foot	High surface adhesion, self-cleaning	Wang et al. (2012); Darmanin and Guittard. (2015); Watson et al. (2015); Sethi et al. (2019)
Cicada wing	Self-cleaning, anti-reflective	Zhang et al. (2006); Xie et al. (2017); Teisala and Butt. (2018); Oh et al. (2019); Román Kustas et al. (2020)
Shark skin	Self-cleaning, underwater drag reduction, self-reparing	Walsh. (1983); Liu et al. (2019); Monfared et al. (2019); Xiang and Liu. (2021)
Penguin feather	Anti-icing, liquid guidance	Wang et al. (2016); Ma et al. (2017); Alizadeh-Birjandi et al. (2020)
Butterfly wings	Self-cleaning, liquid-directed	Zheng et al., 2007; Fang et al., 2008; Tuo et al., 2019)
Spider silk	Water collector	Zheng et al. (2010); Wang et al. (2017); Gustafsson et al. (2018); Si et al. (2018)
Earthworm	Drag reduction, lubrication	Zhao et al. (2018); Xu et al. (2021b); Carmichael. (2021)
Mosquito compound eye	Superhydrophobic, anti-fog	Gao et al. (2007); Wang et al. (2019b); Liu et al. (2021)
Dragonfly wings	Self-cleaning,Superhydrophobic	Nguyen et al. (2013); Nguyen et al. (2014a); Cheeseman et al. (2018)

#### 3.2.1 Gecko feet

Gecko has the ability to crawl on smooth vertical walls, which has aroused researchers’ interest. With the strengthening of research in the past century, the description of the gecko crawling instincts has expanded from macroscopic grasping and suction cup to microscopic Van der Waals forces, which is more and more correct and rigorous. As shown in Table 6 is bionic product with gecko feet as template.

**TABLE 6:** Bionic product with gecko feet as template.

Main materials	Technologies	Advantages	Ref
Choline chloride	Template-free electrodeposition	High adhesion	Li et al. (2020b)
Ethylene glycol			
ZnCl_2_			
Stearic acid			
1H, 1H, 2H, 2 H-perfluorooctane triethoxy silane	Two-step template method	Switching adhesion	Zhang et al. (2021)
Hydrogen peroxide			
Sulfuric acid			
Silicone template			
Castor oil			
Diphenylmethane diisocyanate; Bisphenol An epoxy resin			
Diglycidyl ether			
Dodecylamine			
M- dimethylamine			
N- polyethylene terephthalate			
O- Polyurethane			
Acrylate			
Adhesive			
Polystyrene	Hot pressing	Strong adhesion	Sauer. (2010); Tan et al. (2020)
Aluminum plate	Shear pressing technology		
	Oxygen plasma treatment		
Anodic alumina	—	Strong adhesion	Liu et al. (2012)
4.4′-Oxydianiline			
N,N-dimethylacetamide			
Pyromellitic dianhydride powder; hydrochloric acid			
Fluoroalkylsilane ethanol solution			
Polystyrene			
Alumina membrane	Tmplate-wetting method	Strong adhesion	Jin et al. (2005)
Xylene			
Sodium hydroxide			

Different from the self-cleaning ability of lotus leaf in wet environment, gecko foot has good hydrophobicity, but also has high surface adhesion and self-cleaning performance in dry environment, which provides a direction for the research of dry self-cleaning materials.

Its microscopic state applies to the Gecko state among the five superhydrophobic surface existence states, due to the growth of about half a million micron-level extremely fine bristles on the gecko foot, each bristle end also exists a large number of nanoscale villi branches, which makes the distance between the micro-nano double-order array and the contact surface further reduced and the contact area further increased, so that the sum of the weak Van der Waals forces is sufficient to generate a strong surface adhesion force. The energy barrier for droplet movement increases, so it has the ability to climb walls (Autumn et al., 2000; Autumn et al., 2002; Wang et al., 2012).

As for the mechanism of drying self-cleaning,Xu et al. (2015)showed that geckos used a unique toe-off action in rapid movement, and this dynamic process resulted in a very large instantaneous separation rate of their bristles and contact surfaces. Due to the bristle and shovel-like tentacle system with micro-nano dual-stage structure, the surface adhesion between the foot walls has little to do with the detachment speed, while the detachment force of the microsphere increases with the increase of detachment speed. It is this subtle difference that makes it easy to achieve dry self-cleaning effect during the rapid movement of the gecko. The research results not only provide new design ideas for the long-standing industrial particle manipulation, but also provide a new research direction for the preparation of functional surfaces that can be used repeatedly and have self-cleaning and particle manipulation properties (Kamperman et al., 2010; Liu et al., 2010; Darmanin and Guittard, 2015).

According to the characteristics of geckos, researchers have produced various adhesive materials with high surface adhesion (Li et al., 2011; Liu et al., 2012). In order to design a new type of adhesive film, Zhang et al. (2021) proposed a shape memory film with adhesion to solids and liquids. With high water repellency and low adhesion (about 51 N), this film provides a new idea for the design of different adhesives. Sauer et al. (Tan et al., 2020) prepared nanotube arrays (Eiof∼3 GP) with similar size to gecko bristles from hydrophobic polystyrene, which provided guidance for adhesives designed in wet or underwater environments. In addition, the researchers also used AAO template to prepare multi-scale structure of gecko-like polyimide film. On the basis of stable superhydrophobicity, the film has a high adhesion to water (about 66 μN), and can be used as a manipulator to capture water droplets from a low-adhesion superhydrophobic surface (Liu et al., 2012).

#### 3.2.2 Cicada wings

Compared with the century-old research of gecko, the discovery of super-hydrophobic cicada wings is much later. The Chinese idiom “as thin as a cicada’s wing” is used to describe the extremely small thickness of an object. The scanning electron microscope shows that the thickness of a cicada’s wing is only 8–10 μm, but the self-cleaning and anti-reflection characteristics of cicada’s wings provide another way to discover the superhydrophobic characteristics (Zhang et al., 2006; Dellieu et al., 2014). As shown in Table 7 is bionic product with cicada wings as template.

**TABLE 7:** Bionic product with cicada wings as template.

Main materials	Technologies	Advantages	Ref
Cicada wings	—	Antibacterial	Ivanova et al. (2012)
Polydimethylsiloxane	Template method	Anti-reflection	Liu et al. (2016)
Ethyl orthosilicate			
		Self-cleaning	
Silicon wafer	Deep reactive ion etching	Self-cleaning	Hasan et al. (2015)
C_4_F_8_		Antibacterial	
SF_6_			
O_2_			
Cicada wing	High speed wire electrical discharge machining	Simple, low cost	Liang et al. (2017)
7075 aluminum alloy		Strong mechanical properties	
Molybdenum wire		Environmental friendliness	
Silica microspheres; (Tridecafluoro-1.1,2,2-tetrahydrooctyl)-trichlorosilane;	Self-assembly method	Broadband anti-reflection	Chen et al. (2015)
PET	Chemical etching method		
Ethoxylated trimethylolpropane triacrylate monomer			
Photoinitiator			

Similar to the liquid thin layer at the mouth of pitcher plant, the regular hexagonal micro-nano two-level structure on the surface of cicada wings makes cicada wings have better superhydrophobic performance and self-cleaning ability, especially the micro-nano structure composed of three-dimensional waxy structure is easier to adsorb the air thin layer (Lee et al., 2004; Nguyen et al., 2014b).

Because of its different characteristics, cicada wing is widely used in medical treatment, optoelectronic devices and other fields, mainly due to its antibacterial and anti-reflection properties.

First of all, there are some similarities between cicada wings and lotus leaves in antimicrobial activity (Hasan et al., 2013; Kelleher et al., 2016). In order to limit the spread of infection without antibiotics, Ivanova et al. (2012) used anodization, lithography, micellar lithography and self-assembly to simulate the penetration of nanotube arrays on the surface of cicada wings. They solved the huge losses caused by antibiotic resistance and antibiotic action of pathogens by preparing antibacterial surfaces. The researchers prepared a nanostructured ‘hypersurface’ based on the deep reactive ion etching of silicon wafers. The surface is sustainably antibacterial, kills mammalian cells (mouse osteoblasts), and is used in surgical instruments (Hasan et al., 2015).

In addition, Watson and Watson. (2004) found that compared with plants such as lotus leaves, the hexagonal array of cicada wings has a circular tip extending outward about 150–350 nm. To some extent, this unique structure can be regarded as a kind of gradient refractive index material, which leads to the change of photoimpedance, the decrease of light reflection and the enhancement of antireflectivity (Stoddart et al., 2006; Xie et al., 2017). Inspired by the cicada wing structure, the researchers successfully prepared antireflective films with an average transmittance of 98% and nano-solar cells with strong absorptivity in a wide spectral range. Similarly, Liu et al. (2016) used PDMS to replicate the nano-cone structure of cicada wings to prepare the multi-functional surface of artificial cicada wings. Not only the antireflection effect is outstanding, but also the contact angle of the forward PDMS replica can reach 152°. It has a broad application prospect in many optical equipment.

#### 3.2.3 Penguin feathers

Penguins living in the Antarctic often go to sea to feed, but their feathers do not get wet and are extremely difficult to freeze, which has aroused the interest of researchers. Penguin feathers, as a super hydrophobic material with high ice resistance, which has aroused the interest of researchers and become a hot research object in recent years. In view of the waterproof and ice resistance of penguins, Alizadeh-Birjandi explained the main mechanism of delayed solidification of waterproof materials by developing a heat transfer model, which was extended to general superhydrophobic surfaces (Alizadeh-Birjandi et al., 2020). As shown in Table 8 is bionic product with penguin feathers as template.

**TABLE 8:** Bionic product with penguin feathers as template.

Main materials	Technologies	Advantages	Ref
Steel	One step precipitation polymerization	Effectively delay the icing process	Yang et al. (2016); Latthe et al. (2019)
Hydrogen peroxide		Durable	
Strong acid			
Heptadecafluorodecyl tripropoxy silane			
Body hair of Humboldt cocktail			
1.2,4,5-benzenetetracarboxylic anhydride	Electrospinning	Excellent mechanical strength at low temperature	Wang et al. (2016)
4.4′-diaminodiphenyl ether			
Polyvinylidene fluoride	Electrospinning	Excellent mechanical strength, thermal stability and excellent corrosion resistance	Vicente et al. (2021)
Dimethyl formamide			
Acetone			
Silicon substrate			
Nickel chloride hexahydrate			
Nickel sulfamate tetrahydrate	Lithography	Ice-proof; Wear-resistant	Li et al. (2021c)
Boric acid			
2- ethylhexyl sodium sulfate			
Saccharin sodium hydrate			
Chemical etching method			

(Bormashenko et al. (2012) found that hook-like structures with a diameter of about 3 μm and a spacing of about 20 μm are arranged in an orderly manner on the feather branches parallel to penguin micro-scale and sub-micron feathers. The micro-nano double-stage structure has good hydrophobicity and liquid guiding property, so that the droplets falling on it slide down along the growth direction of the feather.

Further research by Wang et al. (2016) found that the surfaces of feather twigs and feather hooks are not smooth, but lined with grooves with a depth of about 100 nm. These grooves can save air, so that droplets cannot be completely wetted, but exist in Cassie state among five super-hydrophobic surface states, that is, droplets can be regarded as spherical on feather surface, which is easier to slide down and slower in heat dissipation. This multi-stage structure reduces the adhesion between ice and makes penguin feathers have excellent anti-icing performance. In addition, the penguin tail evolved a gland that can secrete oil. Penguin use their beaks to spread oil on feathers, which can play a role in waterproof.

According to the excellent anti-icing and anti-condensation properties of penguin feathers, many applications in heavy industries such as aerospace and ships have been derived. Inspired by the three-dimensional microstructure network of penguin body hair, Wang et al. fabricated a novel polyimide nanofiber film on asymmetric electrodes by electrospinning. The film has good mechanical strength at low temperature (no brittle fracture in liquid nitrogen), which prevents the accumulation of pinning droplets and realizes hydrophobicity. It can be used in the aerospace field to avoid the great danger caused by aircraft icing during flight in extreme weather (Wang et al., 2016). Vicente et al. (2021) used electrospinning technology to prepare functional polyvinylidene fluoride (PVDF) fibers for the excellent hydrophobicity and anti-stickiness of penguin feathers. It can not only prevent the aircraft from drift and resistance caused by atmospheric icing caused by supercooled droplets, but also has excellent mechanical strength, thermal stability and very good corrosion resistance. In the field of ship navigation, researchers used a sprayable mixture of hydrophobic silica nanoparticles embedded in a silica gel matrix to create a bionic superhydrophobic surface that can be used for turbulent drag reduction, thus solving the problem that ships consume a lot of energy to overcome underwater resistance (Golovin et al., 2016). In addition, (Li et al. (2021c) using a simple and potentially low-cost method, a flexible hydrophobic surface was prepared by combining a mechanical durable nickel skeleton with an interconnected microwall array filled with hydrophobic polytetrafluoroethylene (PTFE). Even under the pressure of 0.12 MPa, the prepared surface can remain hydrophobic after more than 1,000 times of linear wear. Compared with the inherent hydrophilic metal surface, the good hydrophobicity also enhances the anti-ice function, and can be used as a multi-functional environmental protection coating in navigation engineering.

#### 3.2.5 Chapter summary

Different super-hydrophobic characteristics of animals are closely related to their living environment. For example, the hydrophobic and anti-icing characteristics of warm feathers are of great significance to the survival of animals in cold regions, while underwater fish have evolved to reduce underwater resistance. People use these different properties and structures to design and manufacture many engineering materials, which provide a reliable guarantee for people’s medical health, aerospace and many other fields.

## 4 Summary and outlook

The hierarchical structure formed by micron-scale papillae and nanoscale wax crystals covering the surface of a lotus leaf, the larger micro-nano double-ordered structure and grooves of rose petal fibres, the geometry of micro-grooves ordered along the same direction in a rice leaf, the bristles and spatula-like tentacle system of the micro-nano double-ordered structure of a gecko foot, the micro-nano structure consisting of regular hexagonal micro-nano two-stage structures and three-dimensional wax structures on the surface of a cicada wing, the micron-scale and The ordered arrangement of the feather branches of sub-micron feathers. All these excellent structures and functions in nature are achieved through multi-level and multi-scale assembly from simple to complex and from disorder to order, which also provides good inspiration for intelligent bionanism in humans. The rich diversity of nature and the adaptive changes of organisms inspire us to think endlessly, and the inventions using the surface hydrophobicity of animals and plants are unique and diverse. From daily necessities to heavy equipment, superhydrophobic materials have attracted people’s unremitting pursuit and exploration for their high performance, low cost and simple preparation process. Based on the principle and concept of superhydrophobic surfaces, this paper mainly introduces the superhydrophobic properties of various animals and plants in nature and their great practical application value, and summarizes the differences and application fields of different superhydrophobic surfaces. Finally, we will put forward a reasonable assumption and plan for the future development prospect of bionic superhydrophobic technology. In view of the achievements and efforts made by our predecessors in constantly exploring the principles and methods of bionic superhydrophobicity, it has laid a solid foundation for us to further develop bionic superhydrophobic materials with simpler, more environmentally friendly materials and lower cost. At present, part of the bionic superhydrophobic technology is gradually changing from the laboratory scale to large-scale industrial production, which has a broad prospect, but the existing problems and shortcomings are also gradually emerging, such as low production efficiency, high production cost, unfriendly to the environment and so on. In this paper, the following ideas are put forward for the future bionic superhydrophobic from natural organism to artificial functional surface:

1) Green, environmentally friendly and sustainable materials make what we are looking for. At present, the Main materials used in the manufacture of superhydrophobic materials are mainly harmful reagents, such as fluorinated superhydrophobic materials, which successfully reduce the surface free energy, but are challenging to the growing environmental and human health problems. We should further develop biodegradable, nontoxic and environmentally friendly new materials into the process of preparing superhydrophobic surfaces, so as to avoid biological pollution and environmental pollution, resulting in irreversible consequences.
2) In light of the fact that the structure and function of these excellent superhydrophobic properties of natural organisms are achieved through multi-level and multi-scale assemblies from simple to complex and from disordered to ordered. Therefore, the development of novel high-performance nanocomposite structures and materials can be achieved by drawing on multiple structures and models.
3) How to make the application materials have sustainable durability has become a big problem. At present, the durability of nanostructure coating on mechanical wear and impact caused by flowing fluid is lower than expected. On the one hand, we need to make some exquisite surface structures, such as micro-nano hierarchical structures or nanostructures, in order to obtain the final superhydrophobic properties. On the other hand, we require the surface to have good surface mechanical properties to meet the requirements of the application. The two are opposing in nature. Therefore, it will be an important research direction in the future that how to achieve a balance or improve its surface mechanical properties on the premise of keeping its surface super-hydrophobic.
4) Compared with the traditional micro-nano processing methods (ion etching, chemical vapor deposition, template method, etc.), femtosecond laser technology has the advantages of high precision, good controllability and applicability to different materials. Therefore, intelligent bionic design with the help of advanced manufacturing technologies and tools such as femtosecond laser machining is also a focus of future research (Yong et al., 2015; Zhang et al., 2020; Fang et al., 2022; Yong et al., 2022; Zhang et al., 2022).
5) The structural and functional design can be coherent and consistent, and the functional design can be considered in conjunction with the natural optical properties of the creature, thus imparting a more aesthetic character.
